# Self-organizing neural maps for multi-modal associations

**DOI:** 10.1186/1471-2202-12-S1-P125

**Published:** 2011-07-18

**Authors:** Mathieu Lefort, Yann Boniface, Bernard Girau

**Affiliations:** 1 LORIA, Campus Scientifique, BP 239, 54506 Vandoeuvre-lès-Nancy Cedex, France

## 

**Figure 1:**
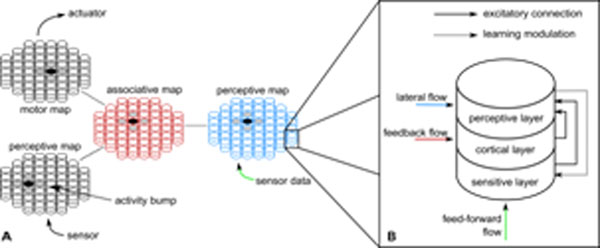
**A** Example of use of our perceptive map model in a multi modal architecture with two sensors and one actuator. **B** Generic structure of one cortical column in a perceptive map.

To move in a complex and dynamic environment, according to the active perception theory, an agent needs to learn the multi modal correlations between its actions and the changes they induce in the environment [1]. Human beings interact with their environment through several distant organs, whose sensory flow processing are influencing each others as, for example, in the Mc Gurk effect [2]. In the functional view of the cortex, each sensory area processes a specific sensory flow and associative areas integrate these flows in a consistent representation of the world that influences in return uni-modal perceptions as for the ventriloquist effect [3]. At a mesoscopic level, the cortex shows a generic structure composed of multi-layer cortical columns. We propose a bio inspired model of perceptive map which, using a continuous and unsupervised learning, self-organizes to map a sensory data flow. Thus, using a spatial competition mechanism, a perceptive map provides an activity bump representing the current perception. In a multi modal architecture, by connecting multiple perceptive maps to an associative map with reciprocal spatially constrained connectivity, the spatial organization of all perceptions have to relax the multi modal constraints (see figure 1A). Thus, this multimodal architecture provides a way to learn multi modal correlations with generalization.

More precisely, each perceptive map consists of generic cortical columns with computing and learning which are local and decentralized (see figure1 B). Sensitive layer receives the feed-forward flow coming from a sensor and uses the BCM learning rule [4]. This synaptic learning rule is based on the hebbian one and is able to autonomously raise a selectivity to one stimulus of the upcoming flow. From a computational point of view, the BCM rule uses a sliding threshold between long term potentiation and long term depression, which has been confirmed by biological evidences [5]. The cortical layer receives feedback influence coming from the associative map, whose activity represents the multi modal perception. The perceptive layer is based on the neural field theory [6] and it receives the sensitive activity modulated by the cortical one. Thanks to the competitive mechanism introduced by the lateral connectivity with a difference of Gaussian shape, an activity bump emerges at the map level, where the activity is the most spatially consistent, representing a consensus between the local sensation and the multi modal constraints.

To obtain a self-organization of the sensitive layer at the map level, the perceptive layer modulates the BCM activity, so that the spatial consistency of the activity bump is propagated to the selectivity organization. We have also introduced an unlearning term in the BCM learning rule in order to forget the current selectivity if it is not consistent with the received modulation. This unlearning mechanism provides plasticity to the self-organization in order to adapt to the multi modal constraints.
